# Advanced glycation end products impair the migration, adhesion and secretion potentials of late endothelial progenitor cells

**DOI:** 10.1186/1475-2840-11-46

**Published:** 2012-04-30

**Authors:** Hong Li, Xiaoyun Zhang, Xiumei Guan, Xiaodong Cui, Yuliang Wang, Hairong Chu, Min Cheng

**Affiliations:** 1 Medicine Research Center, Weifang Medical College, Weifang, Shandong, 261053, P. R. China

**Keywords:** Endothelial progenitor cells, AGEs, Diabetes, Vasoactive substances

## Abstract

**Background:**

Endothelial progenitor cells (EPCs), especially late EPCs, play a critical role in endothelial maintenance and repair, and postnatal vasculogenesis. Advanced glycation end products (AGEs) have been shown to impair EPC functions, such as proliferation, migration and adhesion. However, their role in the regulation of the production of vasoactive substances in late EPCs is less well defined.

**Methods:**

Passages of 3~5 EPCs, namely late EPCs, were cultured with different concentrations (0~500 μg/ml) of AGEs, and the apoptosis, adhesion and migration were subsequently determined. The release of vasoactive substances, such as stromal cell-derived factor-1 (SDF-1), nitric oxide (NO), prostaglandin I_2_ (PGI_2_), plasminogen activator inhibitor-1 (PAI-1), tissue plasminogen activator (tPA), and in addition the activity of superoxide dismutase (SOD), were evaluated by ELISA. At the same time, the gene and protein expressions of CXCR4 were assayed by real-time RT-PCR and western-blot.

**Results:**

AGEs promoted late EPC apoptosis. Moreover, AGEs impaired late EPC migration and adhesion in a concentration-dependent manner. Accordingly, the production of SDF-1 was decreased by AGEs. Although the CXCR4 expressions of late EPCs were up-regulated for AGE concentrations of 50, 100 or 200 μg/ml, a marked decrease was observed for the higher concentration of 500 μg/ml. Furthermore, co-culturing with AGEs decreased the levels of NO, t-PA, PGI_2,_ and the activity of SOD but up-regulated the production of PAI-1.

**Conclusion:**

Our data provide evidence that AGEs play an important role in impairing late EPC functions, which could contribute to the development of vascular diseases in diabetes.

## Background

Diabetes-associated cardiovascular complications, such as atherosclerosis, myocardial infarction and stroke, are among the major causes of patient mortality [1]. It has been shown that loss of endothelial integrity contributes to vascular complications of diabetes [2]. A growing body of evidence indicates that endothelial regeneration and repair do not exclusively rely on the proliferation of local endothelial cells, but also involves bone marrow-derived endothelial progenitor cells (EPCs) [3,4].

EPCs can be isolated from bone marrow, peripheral blood, and umbilical cord blood. Recently, Naito et al even identified an endothelial progenitor/stem-like population located at the inner surface of preexisting blood vessels [5]. Actually, EPCs are heterogeneous and can be classified at least as early and late EPCs. The early EPCs appear within 4 to 7 days while the late EPCs develop after 2 to 3 weeks in ex vivo culture systems. They share some endothelial phenotype but are identified with different morphology, proliferation rate and survival feature [6-8]. Currently, most of the studies have mainly focused on early rather than late EPCs [9,10]. However, late EPCs, which express a variety of endothelial markers and functionally differentiate into mature endothelial cells, seem to also be important in promoting vascular integrity and neovascularization [11,12].

Advanced glycation end products (AGEs), which are produced by the posttranslation modification of proteins via non-enzymatic glycation, accumulate with age and abundantly increase in case of diabetes. They have been shown to be deposited in atherosclerotic lesion, and to promote diabetes-accelerated atherosclerosis [13,14]. Moreover, AGEs impair cell–matrix interactions and growth factor depletion [15]. These evidences suggest that the increased formation of AGEs contributes, at least in part, to the vascular damage in patients with diabetes. Recent studies have shown that AGEs promote EPC apoptosis, inhibit EPC proliferation, and impair EPC functions such as migration, adhesion and tube-forming ability [16,17]. However, growing evidence suggest that EPCs can keep endothelial integrity not only by differentiating into mature vascular endothelial cells, but also by secreting the soluble factors including a number of enzymes like matrix protein, growth factors and cytokines [18]. Although it would be interesting to examine the effects of AGEs on the secretion actions of EPCs, only limited studies have been conducted thus far. The present study therefore aims to evaluate the role of AGEs in regulating the release of vasoactive substances in late EPCs, such as NO, PGI_2_, PAI-1, tPA, together with the activity of SOD. At the same time, we also want to further confirm the effects of AGEs on the apoptosis, migration and adhesion of late EPCs, and to investigate the cellular basis, in particular the role of the SDF-1/CXC4 system.

## Methods

### Bone marrow mononuclear cell isolation and culture

Whole bone marrow was isolated from both the femurs and tibias of Sprague–Dawley rat (150 to 175 g) (Weifang medical College, China). The bone marrow mononuclear cells (MNCs) were fractionated by density gradient centrifugation (Histopaque®-1083, Sigma, USA). MNCs were plated on dishes precoated with 5% fibronectin (Roche, Germany), and were maintained in complete EGM-2 medium (supplemented with EGM-2 bullet kit, including 5% fetal calf serum, recombinant rat VEGF, recombinant human bFGF, Invitrogen, USA). After 4 days in culture, unattached cells were removed by washing with PBS, after which fresh medium was added. Endothelial colonies subsequently appeared (on average 1 colony/10^7^ or 10^8^ plated mononuclear cells). Highly proliferative endothelial cells grew out from these colonies which then formed a confluent monolayer. Cells under passage 3~5, namely late EPCs, were used for the present study [19]. All animal protocols were approved by the local ethics committee at Weifang Medical College.

### EPC identification

After 7 days of culture, EPCs were characterized by the uptake of 1,1′-dioctadecyl-3,3,3′,3′- tetramethylindo-carbocyanine–labeled acetylated low density lipoprotein (Dil-acLDL, Molecular probes, USA), and by fluorescein isothiocyanate labeled Ulex europaeus agglutinin (FITC- UEA-1, Sigma, USA) staining. In short, the adherent cells were first incubated with Dil-acLDL (2 μg/ml) for 1 h, after which they were fixed in 2% paraformaldehyde for 10 min, and then stained with FITC-UEA-1 (10 μg/ml) for 1 h. After the staining, the samples were viewed with inverted fluorescence microscope (Leica, Germany). The early EPCs were moreover confirmed by the expressions of CD133 (eBioscience, USA, dilution 1:50) and Sca-1(Becton Dickinson, USA, dilution 1:50). Cells under passage 3~5, namely late EPCs, were analyzed by FACS (Becton Dickinson, USA) for the expressions of vWF (Sigma, USA, dilution 1:200), VEGFR2 (eBioscience, USA, dilution 1:100), VE-cadherin (Becton Dickinson, USA, dilution 1:400) and PECAM-1(eBioscience, USA, dilution 1:100). The capability of EPCs to form capillary-like tubes in vitro was assessed on Matrigel.

### FACS analysis of apoptosis

Late EPCs were treated with AGEs (Biovision, USA) for 24 h. The range of AGE concentrations used in all experiments was at a dose of 0~500 μg/ml which is representative of the plasma concentrations of AGEs found in diabetic patients [20]. Approximately 1 × 10^6^ cells were double-stained with Annexin V-FITC and propidium iodide (PI) by using apoptosis detection kit (Becton Dickinson, USA) according to the manufacturer’s instructions. Apoptotic cells (Annexin V ^+^/PI^-^) were detected by FACS. The apoptotic percentage analysis was performed using Cell-Quest software (Becton Dickinson, USA).

### EPC migration assay

The migratory function of EPCs was evaluated by a modified Boyden chamber (Costar, Cambridge, MA, USA) assay. Briefly, after incubation with serum-free medium for 6 h, EPCs were isolated with trypsin/EDTA, and then incubated with serum-free medium containing AGEs. A total of 4 × 10^4^ EPCs were placed in the upper chamber while medium with VEGF (50 ng/ml) was placed in the lower chamber. The assays were conducted over a 24 h incubation period at 37°C in an incubator equilibrated with 5% CO_2_. The membrane was then washed gently with PBS, and fixed with 4% paraformaldehyde. Non-migrating cells were gently removed with cotton balls from the upper side of the membrane which was then stained by using 4′,6-diamidino-2-phenylindole (DAPI). The migration of EPCs was evaluated by counting the migrated cells in six random high-power (100×) microscope fields/well.

### EPC adhesion assay

EPC adhesion assay were performed as previously described [21]. After being treated with different concentrations of AGEs for 24 h, EPCs were washed with PBS, and then gently detached with 0.25% trypsin/ EDTA. After centrifugation and resuspension with serum-free medium, equal cell numbers were seeded on fibronectin coated culture dishes, and incubated for 30 min at 37°C. Adherent cells were counted independently in six random high-power (×100) microscope fields (HPF)/well by three observers unaware of the treatments.

### Assay of the expressions of CXCR4 of EPCs by real-time RT-PCR and western blot

Total cellular RNA was isolated with TRIzol reagent (Invitrogen, USA) and reverse-transcribed to cDNA using the SYBR® PrimeScript® RT-PCR Kit (Takara, Japan) at 37°C for 15 min. Gene expressions were evaluated by SYBR® Premix Ex Taq™ (Takara, Japan). Rat CXCR4 was amplified with the sense primer 5′-AGC AGG TAG CAG TGA CCC TCT GA-3′and the anti-sense primer: 5′-GAA GCA GGG TTC CTT GTT GGA GT- 3′. GAPDH (sense primer: 5′-GGC ACA GTC AAG GCT GAG AAT G- 3′, anti-sense primer: 5′-ATG GTG GTG AAG ACG CCA GTA- 3′) was used as a housekeeping gene, in order to normalize the expression target gene. All primers were used at a final concentration of 0.4 μmol/L. The thermal cycling conditions were as follows: 30 seconds at 95°C for pre-denaturation, 40 cycles for 15 seconds at 95°C for denaturation, 1 minute at 59°C for annealing, and 10 seconds at 72°C for elongation. At the end of each cycle, the fluorescence emitted by the SYBR Green I was measured. After completion of the cycling process, samples were immediately subjected to a temperature ramp for melting curve analysis. The relative gene expression was analyzed by the 2^-DDCt^ method [22].

Total cellular protein was extracted in 150 μl of 1 × SDS loading buffer (62.5 mmol/L Tris–HCl pH 6.8, 2% SDS, 10% glycerol, 50 mmol/L DTT, 0.1% bromphenol blue) in the presence of 0.1% EDTA-free protease inhibitor cocktail. Protein was quantified using the bichoninic acid assay (BCA; Pierce Biotechnology, Rockford, IL, USA) according to the manufacturer’s instructions. Equal amounts of protein (50 μg) were separated through a 12% SDS-PAGE, and transferred to a PVDF membrane. Membranes were blocked in 5% milk-TBST, followed by overnight incubation with the primary antibodies against CXCR4. The membranes were then washed with TBST, and incubated with secondary antibody conjugated to HRP (Santa Cruz, USA, dilution 1:1000). Immunoreactive bands were visualized by chemiluminenscence (ECL Amersham Pharmacia).

### Assay of secretion actions of EPCs

To measure the secretion actions of EPCs, the cultures were washed and re-fed with equal amounts of serum-free medium containing different concentrations of AGEs for 24 h. The medium was collected and 10 × concentrated by centrifugation for 20 min at 5000 × g at 4°C using Ultrafree-4 centrifugal filter tubes with Biomax-5 membrane(Millipore, USA). The levels of SDF-1, NO, t-PA, PAI-1, PGI_2_, and the activity of SOD in the cell culture supernatants were measured by sandwich enzyme-linked immunosorbent assay (ELISA, RD, USA) according to the manufacturer’s instructions. Briefly, standard or testing sample (50 μl) was added to the 96-well plate containing immobilized monoclonal antibodies against a kind of factor. After mixing by shaking gently, the plate was incubated at 37°C for 30 min. The wells were then washed five times, and incubated with HRP-conjugate reagent at 37°C for 30 min. Afterwards, chromogen solution A and chromogen solution B were added to each well. The reaction was terminated after 20 min by adding the stop solution. Blank well was taken as zero, and the optical density (OD) was measured with an enzyme-linked immunoabsorbent assay reader (Bio-Rad Laboratories, USA).

### Statistical analyses

Unless otherwise indicated, results are reported as mean ± SE from at least 4 independent experiments. Statistical analyses were performed by one-way ANOVA, followed by Tukey-Kramer post hoc test for multiple comparisons, and p < 0.05 was considered to be statistically significant. All data were analyzed using SPSS software (version 15.0; SPSS, Chicago, IL, USA).

## Results

### Characterization of bone marrow-derived late EPCs

**Figure 1:**
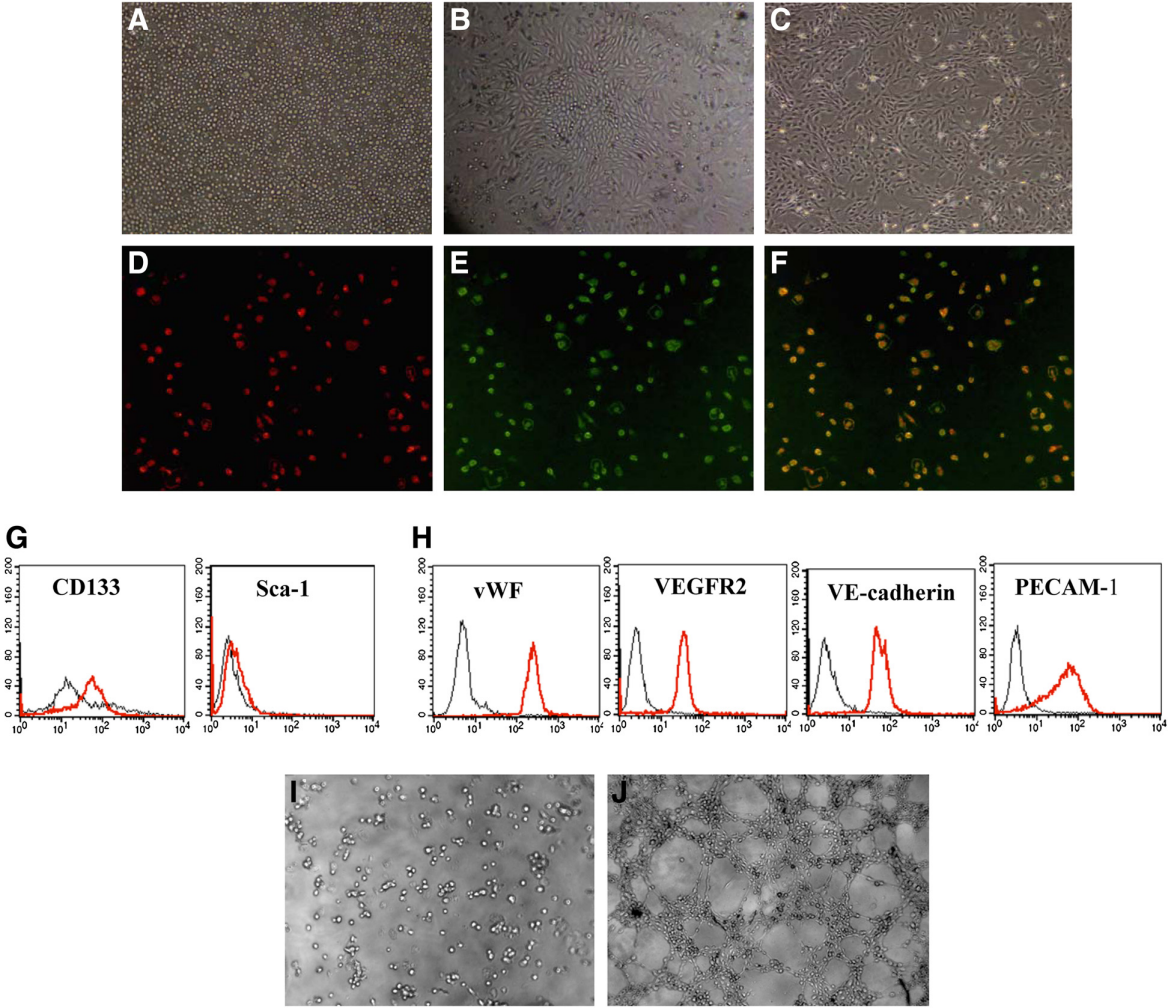
**Characterization of EPCs derived from bone marrow. A:** MNCs were isolated and plated on fibronectin-coated culture dish on the first day (100×). **B:** Seven days after plating, adherent early EPCs with spindle shape formed clones (100×). **C:** After 3~4 weeks, the third-fifth passage cells (late EPCs) showed cobblestone-like morphology (100×). **D**-**F:** Most early EPCs were shown to simultaneously endocytose DiI-acLDL (red) and bind fluorescein isothiocyanate UEA-1 (lectin) (100×). **G:** FACS analysis showing the expressions of CD133 and Sca-1 in early EPCs. Plots show isotype controls (black) *vs.* specific antibody staining (red). **H:** FACS analysis showing the phenotype of late EPCs using several markers: vWF, VEGFR-2, VE-cadherin and PECAM-1. Plots show isotype controls (black) *vs* specific antibody staining (red). **I**-**J:** Representative images of tubuli like structures formed on Matrigel by early and late EPCs(100×).

The bone marrow-derived MNCs that initially seeded were round (Figure 1A). The distinct colonies formed after 48 h, reaching a peak on the seventh day. The colonies of early EPCs appeared with the round cells in the centers and the typical spindle cells at the peripheries after 7 days (Figure 1B), and were identified as double-positive for Dil-acLDL uptake and lectin binding affinity (Figure 1D, E, F). Further FACS analysis revealed that the respective expressions of CD133 and Sca-1 were around 24.5% and 3.3% in early EPCs (Figure 1G). After 3~4 weeks, the 3~5 passage cells (late EPCs) showed characteristic homogeneity and cobblestone-like morphology similar to mature endothelial cells as previously reported (Figure 1C) [23]. Moreover, the majority of the cells expressed endothelial-specific markers: vWF, VEGFR-2, VE-cadherin and PECAM-1 (Figure 1H) [10]. The angiogenic property of the EPCs was also studied. As shown in Figure 1I and Figure 1J, late, but not early, EPCs successfully formed tubuli like structures on Matrigel.

### Effects of AGEs on late EPC apoptosis

**Figure 2:**
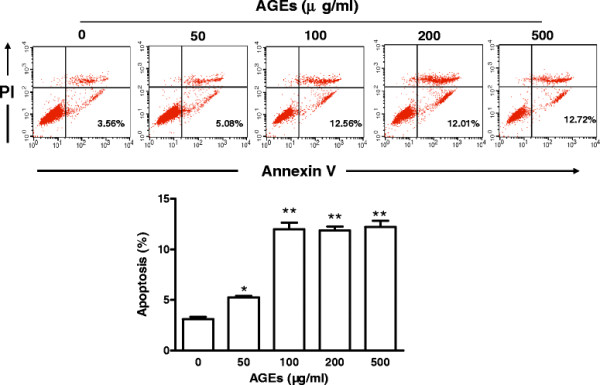
**Effects of AGEs on late EPC apoptosis. A:** Late EPCs were incubated with different concentrations of AGEs (0, 50, 100, 200 and 500 μg/ml) for 24 h. Harvested cells were stained with Annexin V FITC and PI, and quantified by FACS. Right lower quadrant means Annexin V positive and PI negative. Right superior quadrant means Annexin V and PI positive. Left superior quadrant means Annexin V negative and PI positive. Left lower quadrant means Annexin V and PI negative. Annexin V-positive and PI-negative cells were defined as apoptotic cells. **B:** The proportion of apoptotic cells. Data represent the mean ± SE of four different experiments. *p < 0.05, **p < 0.01 *vs.* control.

Late EPCs were incubated with different concentrations of AGEs (0, 50, 100, 200 and 500 μg/ml) for 24 h. As shown in Figure 2, AGEs induced apoptosis in late EPCs. The number of apoptotic cells (Annexin V ^+^/PI ^-^) was observed to increase with higher concentrations of AGEs, reaching a peak at 100 μg/ml, and subsequently saturating to a fixed value.

### Effects of AGEs on late EPC migration, adhesion and SDF-1/CXCR4 system

**Figure 3:**
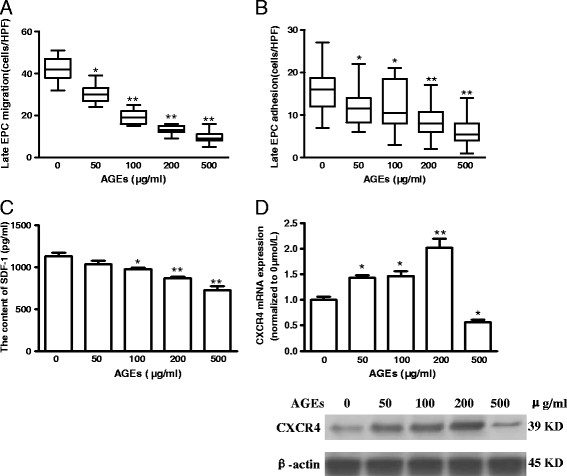
**Effects of AGEs on late EPC migration, adhesion and SDF-1/CXCR4 system.** Late EPCs were treated with different concentrations of AGEs (0, 50, 100, 200, and 500 μg/ml) for 24 h. **A:** The modified Boyden chamber assay was used with VEGF as a chemoattractive factor for late EPC migratory function. The migrated cells were stained with DAPI and counted under microscope. **B:** EPCs with equal cell numbers were seeded on fibronectin coated culture dishes and incubated for 30 min at 37°C. Adherent cells were counted for late EPC adhesion function. **C:** The supernatants were collected, and the content of SDF-1 was analyzed by ELISA. **D:** The CXCR4 mRNA expressions were determined using real time quantitative RT-PCR. **E:** Cell lysates were resolved on 12% SDS-PAGE, followed by transfer to PVDF membrane. Western blot was carried out with specific antibody for CXCR4, and each band was detected by the ECL reagent. In addition, the β-actin was analyzed as loading control and protein. Data represent the mean ± SE of four different experiments. *p < 0.05, **p < 0.01 *vs.* control.

To examine the effects of AGEs on late EPC migration, cells were treated for 24 h with different concentrations of AGEs in the upper compartment of a Boyden chamber, the lower compartment of which contained EBM-2 medium with VEGF. The AGEs were observed to inhibit late EPC migration in a concentration-dependent manner, with a 4-fold decrease at the maximal concentration tested (500 μg/ml, Figure 3A). Moreover, late EPC adhesion on FN was also significantly reduced in a concentration-dependent manner with adherent cells at less than 38% of the control value when applied to the maximal dose of AGEs (500 μg/ml, Figure 3B). To determine whether the impaired migration and adhesion observed in AGE-stressed EPCs was related to the SDF-1/CXCR4 system, the release of SDF-1 was measured by ELISA. At the same time, the mRNA and protein expressions of CXCR4 were assessed by real-time RT-PCR and western blot, respectively. The AGEs were found to induce a decrease in the SDF-1 secretion in a dose-dependent manner (Figure 3C). However, EPCs treated with 50, 100 and 200 μg/ml of AGEs displayed higher mRNA and protein expressions of CXCR4 than those in the untreated sample. Interestingly, 500 μg/ml of AGEs markedly decreased the CXCR4 mRNA and protein levels of late EPCs (Figure 3D, E).

### Effects of AGEs on the productions of the vasodilators, NO and PGI_2_, in late EPCs

**Figure 4:**
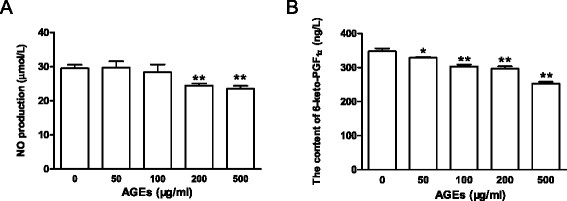
**Effects of AGEs on the productions of the vasodilators, NO and PGI**_**2**_**, in late EPCs.** Late EPCs were incubated in medium containing AGEs of concentrations between 0 ~ 500 μg/ml for 24 h, and the cultured media were then collected. **A:** The amount of NO was determined by ELISA. **B:** The production of 6-keto-prostaglandin F_1α_ (6-keto-PGF_1α_), the PGI_2_ stable degenerative product, was determined by ELISA. Data represent the mean ± SE of four different experiments. *p < 0.05, **p < 0.01 vs. control.

To investigate the effects of AGEs on the productions of the vasodilators in late EPCs, cells were incubated for 24 h in medium containing different concentrations of AGEs (0 ~ 500 μg/ml). The cultured media were collected, and the amounts of NO and PGI_2_ released from the late EPCs were determined by ELISA. Given that PGI_2_ has a short half-life (<20 minutes at physiological pH), levels of 6-keto-prostaglandin F_1α_ (6-keto-PGF_1α_), the PGI_2_ stable degenerative product, were quantified to evaluate the PGI_2_ secretion from late EPCs. The results show that the incubation of late EPCs with higher concentrations of AGEs (200 and 500 μg/ml) significantly decreased the production of NO, compared with that in the control medium. For the lower concentrations of AGEs (50 and 100 μg/ml), however, no such decrease was observed (Figure 4A). The production of 6-keto-PGF_1α_ by AGE-stressed EPCs was reduced in a dose-dependent manner. (Figure 4B)

### Role of AGEs on the activity of SOD in late EPCs

**Figure 5:**
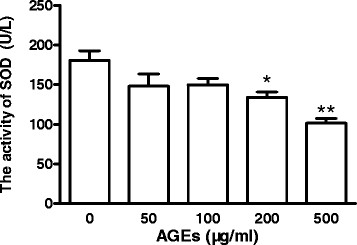
**Role of AGEs on the activity of SOD in late EPCs.** Late EPCs were stimulated for 24 h with varying concentrations of AGEs (0 ~ 500 μg/ml), and the cultured media were subsequently collected. The SOD activity of late EPCs were assessed by ELISA. Data represent the mean ± SE of four different experiments. *p < 0.05, **p < 0.01 vs. control.

The bioactivity of SOD was measured in late EPCs. As shown in Figure 5, incubation of cells with higher concentrations of AGEs decreased the SOD activity in EPCs as compared to the control medium. The SOD activity of EPCs was reduced by 25.9% and 43.7% in AGE concentrations of 200 and 500 μg/ml, respectively.

### Influence of AGEs on PAI-1 and t-PA secretions from late EPCs

**Figure 6:**
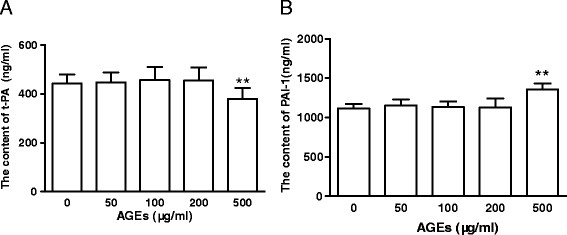
**Influence of AGEs on PAI-1 and t-PA secretions from late EPCs.** Late EPCs were exposed to AGEs at concentrations of 0 ~ 500 μg/ml for 24 h, after which they were tested for t-PA (**A**) or PAI-1 (**B**) production by ELISA. Data represent the mean ± SE of four different experiments. **p < 0.01 *vs.* control.

Since the balance between PAI-1 and tPA determines the fibrinolytic activity, we also investigated the effects of AGEs on the productions of PAI-1 and tPA from late EPCs. To this end, late EPCs were exposed to AGEs at concentrations of 0 ~ 500 μg/ml for 24 h, after which they were tested for PAI-1and tPA productions by ELISA. Incubation of late EPCs with 500 μg/ml of AGEs led to a significant decrease in the production of t-PA. However, no statistically significant decrease was observed for low concentrations of AGEs (Figure 6A). In contrast, incubation of late EPCs with 500 μg/ml AGEs led to an increase in the production of PAI-1 (Figure 6B).

## Discussion

AGEs,as a result of chronic hyperglycemia in diabetic patients, have been identified as one of the key risk factors in diabetic-accelerated atherosclerosis [24]. Cell activation, proinflammatory cytokine release and oxidative stress, induced by the interaction of AGEs with their receptors, may lead to endothelial injury and dysfunction [25,26]. Growing evidence suggests that EPCs, mobilized from bone marrow and other sites, contribute to the structural integrity of the vasculature, and also restore endothelial dysfunction [3]. Recently, Palombo et al [27] have reported that a significant reduction in the number of EPCs is related to carotid intima-media thickness, an induction of early atherosclerosis in diabetes mellitus. Moreover, the frequency of EPCs in DM patients is negatively correlated with the levels of hemoglobin AIc (HbAIc) [28], and DM patients with satisfactory glycemic control (defined by Hemoglobin A1c, HbA1c < 6.5%) have been found to have significantly higher circulating EPC counts [29]. On the contrary, reduction in circulating and soluble form of AGEs by azelnidipine [30] may have beneficial effects on glucose tolerance, insulin sensitivity, the inflammatory state, and number of EPCs [31].

Sun et al [32] have reported that the receptors of AGEs (RAGE) are expressed on EPCs at a low level in normal conditions, and that AGEs significantly increase the RAGE expression, and promote EPC apoptosis in a dose-dependent manner (50~300 μg/ml). In agreement with those findings, we have here shown that AGEs induce apoptosis in late EPCs, with the apoptosis rate, however, reaching peak at a concentration of 100 μg/ml, after which it settles at a constant value even when the AGE concentration is increased further. One possible explanation for the differences between the observed relationship between apoptosis rate and AGE concentration could be the different types of EPCs used in these studies. Late EPCs were used in our study, while Sun et al. used early EPCs in their experiments.

Previous investigations have also demonstrated that AGEs impair EPC functions, such as migration, adhesion and tube formation [16,17]. Our results further confirm that AGEs inhibit migration and adhesion in late EPCs. It is then natural to ask what the mechanisms behind this might be. SDF-1 and its transmembrane receptor CXCR4 play a pivotal role in regulating the trafficking of immature stem cells [33,34]. Enhanced SDF-1 expression and production is essential for the migration and adhesion of stem cells [35]. It has been shown that ex vivo exposure of EPCs to SDF-1 induces cell migration and adhesion [36]. Moreover, Smadja et al [37] have demonstrated that the angiogenic ability of EPCs may be mediated by an autocrine mechanism involving SDF-1/CXCR4. To characterize the role of the SDF-1/CXCR4 system in the AGE-induced decrease of the migration and adhesion of late EPCs, we quantified the expressions of SDF-1 and CXCR4. We have observed here that AGEs induce a reduction in SDF-1. Intuitively, one could then expect that AGEs might result in a decrease in CXCR4 as well. In our study, however, we found that AGEs led to the CXCR4 overexpression on the late EPCs, with the overexpression increasing as a function of the AGE concentration only up to a certain point (200 μg/ml), whereas beyond this, at a very high concentration (500 μg/ml), the CXCR4 expression levels were seen to decline. Thus the elevated CXCR4 expression may account for a commentary response to the defective SDF-1 in the AGE stressed-EPCs while a high concentration of AGEs can destroy the intrinsic commentary ability of late EPCs.

Due to the fact that the release of vasoactive factors by EPCs also contributes to the vasculature integrity and homeostasis [18], we explored in addition the influence of AGEs on late EPC secretion functions. The results show that AGEs cause a reduction in NO, PGI_2_ and tPA. Furthermore, AGEs were identified to decrease the SOD activity of EPCs. At high concentrations, however, the AGEs were seen to increase the secretion of PAI-1 by EPCs.

NO is a major vasodilator and a key survival factor of the endothelium. The reduction in NO production is possibly contributing to the development of endothelial dysfunction and atherosclerosis in diabetes mellitus [38]. Ozuyaman and colleagues have demonstrated that NO can stimulate EPC mobilization from bone marrow stem cell niches to the peripheral circulation [39]. Moreover, it has also been shown that the treatment of EPCs from diabetes patients with an NO donor drug normalizes their migration [40], and that SDF-1α restores EPC homing to wounds in diabetic mice through an NO-dependent mechanism [41]. Urao et al have reported that transplantation of autologous EPCs overexpressing eNOS in injured vessels enhances the vasculoprotective prosperities of reconstituted endothelium [42]. Therefore, NO which is produced by the EPCs themselves may create a favorable and optimal environment in regulating their functions. Liang et al [21] reported that the expression of phosphorylated-eNOS is reduced by AGEs in EPCs. In line with this finding, our results show that a 24 h incubation with AGEs significantly decreases bioavailable NO of late EPCs, which would be one of the determinants of vascular damage in diabetic patients.

In addition to NO, PGI_2_ is not only a potent vasodilator contributing to the protection and maintenance of homeostasis in vasculature but also an inhibitor of platelet activation. It has been shown that PGI_2_ has a direct effect on EPC functions in an autocrine or paracrine manner through an NO-dependent mechanism, and EPCs from prostacyclin receptor (IP−/−) mice fail to promote re-endothelialization and vessel repair [43]. In the present study, our results show that AGEs impair PGI_2_ release in cultured EPCs, which may be one of the reasons why the levels of PGI_2_ is low in the early stages of diabetes [44]. Furthermore, decreased PGI_2_ has been linked to platelet hyperaggregability, increased adhesiveness, and increased release of PGH2/TXA2 in diabetic patients [45].

It has become evident that the interaction between NO and O_2_^-^ is important in the development of endothelial dysfunction [46]. EPCs have high intracellular expression levels of SOD, which can protect them against oxidative stress [47].Thum et al have reported that increased NAD(P)H oxidase activity results in increased O_2_^-^ generation and reduced NO bioavailability because O_2_^-^ inactivates NO and uncouples eNOS in EPCs [48]. Moreover, the increased O_2_^-^ is closely associated with the reduced extracellular SOD activity [49]. Thus the addition of SOD may scavenge O_2_^-^, increase NO bioavailability and/or prevent the uncoupling of eNOS in glucose-stressed EPCs [50].We have here shown that AGEs dose-dependently inhibited SOD activity, which may account for AGEs decreasing bioavailable NO of late EPCs. Thus, AGEs may increase the risk of developing cardiovascular diseases by decreasing the SOD activity of EPCs.

Clinical and experimental studies suggest that thrombus formation and plaque rupture are other major aspects of cardiovascular diseases [51]. t-PA is one of the most important fibrinolytic substances which regulates fibrinolytic activity and prevents thrombus formation. Serving as the primary inhibitor of t-PA, PAI-1 plays a critical role in the regulation of fibrinolysis. The balance between these proteins is known to control the development of thrombosis. A recent study has shown that EPCs can also produce t-PA, and that the amount secreted by EPCs is comparable to that secreted by mature endothelial cells [52]. Thus we have here also studied the effects of AGEs on the secretion of t-PA and PAI-1 from late EPCs. We have shown that incubation of late EPCs with high concentrations of AGEs leads to a significant decrease in the production of t-PA, while increasing the production of PAI-1. The different effects of AGEs on t-PA and PAI-1 might lead to an imbalance between them, inducing EPCs to become procoagulative and hypofibrinolytic.

## Conclusion

Taken together, the present study demonstrates that AGEs exert deleterious effects on EPC functions, such as migration, adhesion and secretion action. It provides evidence that diabetes via AGEs may contribute to the functional defects of the endothelium in pathological situations by causing the dysfunction of EPCs. Furthermore, these findings give further insight into the complex cellular mechanisms of EPCs for impaired vascular repair and neovasculogenesis in diabetic patients.

## Abbreviations

EPCs = Endothelial progenitor cells; AGEs = Advanced glycation end products; SDF-1 = Stromal cell-derived factor-1; NO = Nitric oxide; PGI2 = Prostaglandin I2; PAI-1 = Plasminogen activator inhibitor-1; tPA = Tissue plasminogen activator; SOD = Superoxide dismutase; Dil-acLDL = 1,1′-dioctadecyl-3,3,3′,3′- tetramethylindo- carbocyanine–labeled acetylated low density lipoprotein; FITC-UEA-1 = Fluorescein isothiocyanate labeled Ulex europaeus agglutinin; vWF = Von Willebrand factor; VEGFR2 = Vascular endothelial growth factor receptor 2; VE-cadherin = Vascular endothelial cadherin; PECAM-1 = Platelet endothelial cell adhesion molecule-1.

## Misc

Hong Li and Xiaoyun Zhang contributed equally to this study.

## Competing interests

The authors declare that they have no competing interests.

## Authors’ contributions

HL, XYZ and MC were the principal investigators, involved in designing the study and writing the manuscript. XMG coordinated the study and performed statistical analyses. XDC, YLW and HRC were involved in cell culture, western blot and writing parts of the manuscript. All authors participated in writing the final version of the manuscript.
